# Identification of risk factors for ewe mortality during the pregnancy and lambing period in extensively managed flocks

**DOI:** 10.1186/s12917-023-03822-x

**Published:** 2023-12-06

**Authors:** K. J. Flay, A. S. Chen, D. A. Yang, P. R. Kenyon, A. L. Ridler

**Affiliations:** 1 Department of Veterinary Clinical Sciences, City University of Hong Kong, 31 To Yuen St, Kowloon, Hong Kong SAR China; 2 College of Veterinary Medicine, Nanjing Agricultural University, 1 Weigang, Nanjing, 210095 China; 3 School of Agriculture and Environment, Massey University, Private Bag 11-222, Palmerston North, New Zealand; 4 School of Veterinary Science, Massey University, Private Bag 11-222, Palmerston North, New Zealand

**Keywords:** Ovine, Sheep, Death, Wastage, Body condition score (BCS), Weight, Reproduction, Litter size

## Abstract

**Background:**

Ewe mortality during pregnancy and lambing is an issue for sheep producers globally, resulting in reduced productivity and profitability, compromised ewe welfare, and poor consumer perception. Despite these negative consequences, there was little investigation into factors associated with ewe death during this time. Therefore, this study aimed to assess associations between ewe body condition score (BCS), weight, reproductive parameters, and risk of mortality during pregnancy and lambing.

**Methods:**

Four cohorts from three commercial New Zealand farms participated, with 13,142 ewe lambs enrolled and followed over time. Data were collected for five consecutive lambings. Visits aligned with key on-farm management times, specifically: prior to breeding, at pregnancy diagnosis (PD), prior to lambing (set-stocking), and, at weaning of their lambs. At each visit, ewes were weighed, BCS assessed and reproductive status was recorded when relevant (litter size at PD and lactation status after lambing). Ewes that died or were culled were recorded, and any ewes that were absent from consecutive visits were presumed dead. Logistic regressions were developed to assess the relationship between weight and BCS at each visit, PD result (single or multiple-bearing) and lactation status (wet or dry) in each year, and, risk of mortality during the pregnancy and lambing period in each year.

**Results:**

In the PD to weaning period, mortality incidence ranged from 6.3 to 6.9% for two-tooth (18-months-old at breeding) to mixed-age (54-months-old at breeding) ewes. For ewe lambs (7 to 8-months-old at breeding), mortality was 7.3% from set-stocking to weaning. Heavier ewe lambs at PD were less likely to die during lambing (OR: 0.978, p = 0.013), as were those with greater set-stocking BCS. In subsequent years, BCS was a predictor of ewe death, with odds of mortality greatest for ewes < BCS 2.5. Additionally, for poorer BCS ewes, increasing weight reduced risk of mortality, but there was no impact of increasing weight in greater BCS ewes.

**Conclusions:**

This study identified risk factors associated with ewe mortality during the pregnancy and lambing period. Flock owners can use these to either cull at-risk ewes or proactively intervene to reduce likelihood of mortality, thereby improving flock productivity, profitability and welfare.

**Supplementary Information:**

The online version contains supplementary material available at 10.1186/s12917-023-03822-x.

## Introduction

Ewe mortality is a known issue in sheep flocks globally [1, 2], resulting in reduced productivity and profitability [3] and compromised sheep welfare [4, 5]. Recent studies have reported increased mortality incidence and/or risk of death during the lambing period [4, 6–8]. In instances where cause of ewe death during this period is investigated it is typically caused by a condition or disease directly linked to pregnancy and parturition; for example, dystocia or pregnancy toxaemia [9–12].

In extensively managed pasture-based flocks recently reported ewe mortality rates during lambing range from 0.5 to 13.7% [8, 9, 13]. However, studies had varying observation periods and methodologies; [9] and [13] relied on farmer reported data and estimated ewe mortality on a number of farms, while [8] reported researcher collected data limited to a single farm. There are likely inaccuracies when relying on farmer-reported data, with [14] finding that > 50% of farmers underestimated on-farm ewe mortality. In general, ewe mortalities appear to be poorly recorded in extensive sheep flocks [2, 14].

Despite the importance of ensuring ewes survive and rear their offspring there has been little research into causes and risk factors for on-farm ewe mortality during lambing [8, 9, 11, 12]. Productivity parameters such as body condition score (BCS), weight, litter size and previous reproductive performance are particularly relevant to investigate as risk factors, as they are already recorded by many farmers and/or are measures that are relatively straightforward to implement in extensively managed flocks [2, 15, 16]. Additionally, these factors have been shown to influence annual ewe mortality incidence/risk [5, 7] and are risk factors for pregnancy and parturition related disease conditions, so it is likely they will also impact ewe mortality over lambing.

The aims of this study were to assess the associations between ewe BCS, weight, litter size and previous reproductive performance, and risk of ewe mortality during the pregnancy and lambing period as ewes aged from ewe lambs to mixed-age ewes, in a sample of commercial pasture-based flocks.

## Materials & methods

### Farms and animals

This study used data collected from 13,142 ewes that were managed as part of three large privately owned commercial flocks (Farm A 2010-born, n = 3717; Farm A 2011-born, n = 4609; Farm B, n = 3998; Farm C, n = 818) in North Island, New Zealand, pasture-based sheep and beef farms. Farm A ewes were a semi-stabilized composite breed consisting of Coopworth and East Friesian genotypes, while ewes from Farm B and Farm C were Romney. Data were collected as the ewes aged from replacement ewe lambs (7–8 months old at time of enrolment) to 6-years-old (Farms A and B) or to 3-years-old (Farm C) during the period 2011–2017.

Farms were convenience sampled based on large flock sizes, existing use of farm management practices such as body condition scoring, pregnancy diagnosis (PD), and electronic identification (EID) tags (needed for data recording), combined with a willingness to commit to a longitudinal lifetime study (≥ 5 years). Detailed information regarding the selection, reproductive and general management and lifetime of these ewes has been described by [7], as part of a longitudinal lifetime study of ewe wastage. During the lambing period ewes from Farm A were observed by farm staff every 2–3 days, while on Farm B and Farm C they were observed daily. Any obvious problems such as dystocia and vaginal prolapse were resolved; however, their incidence was not recorded by the farm staff as per normal practice for each of these farms.

### Data collection

Researchers visited each farm at four key management times in each year: prior to breeding (mating), at PD (mid-pregnancy), at set-stocking (where this occurred within four weeks of lambing; pre-lambing) and at weaning. A summary of the exact calendar timing of each data collection visit is available in [7]. At each visit, body condition score (BCS) and weight were recorded for all study ewes. BCS was assessed by palpation of the soft tissues over the lumbar region, using a 1–5 scale (1 = thin; 5 = obese), assessed to the nearest 0.5 of a BCS [17]. At the PD visit, the researchers also recorded the PD result for each study ewe, while at the weaning visit the lactation status (wet or dry) was recorded based on data provided by the flock manager. A ewe that was lactating (wet) was assumed to have at least one live lamb, while those that were not lactating (dry) were assumed to have either had a pregnancy loss, abortion, or their offspring had died during the perinatal period. Cull and mortality data were recorded at each visit, with detailed information regarding the incidence of ewe culling and mortality throughout their lifetime available in [7].

### Data inclusion

As this study aimed to investigate risk factors associated with ewe mortality over the pregnancy and lambing period, only ewes that were retained in their respective flocks for lambing in each year were eligible for inclusion in the analyses for each ewe age (i.e., ewes that were culled or died before the period of interest were excluded). The time period of interest was set-stocking to weaning for the ewe lamb age group, and PD to weaning for all other ages (two-tooth, four-tooth, six-tooth and mixed age). In each year, eligible ewes were allocated a “lambing fate” reflecting if they died (“dead”), or survived (“alive”), during the period of interest. “Dead” included ewes that died on farm and those who went missing during the time-period. Overall, lambing fates were allocated for 11,143 ewe lambs (dead = 819; alive = 10,324), 8,912 two-tooths (dead = 561; alive = 8,351), 6,627 four-tooths (dead = 458; alive = 6,169), 5,126 six-tooths (dead = 342; alive = 4,784) and 3,840 mixed-age ewes (dead = 240; alive = 3,600).

### Statistical analyses

Data was stored in Microsoft Excel, organised according to each ewe’s unique EID tag number. Prior to analyses the data was segregated into subsets according to age of ewe and therefore pregnancy and lambing period of interest e.g., the ewe lamb analysis included only data from when the ewes were ewe lambs vs. the four-tooth analyses considered data from prior years (ewe lamb and two-tooth) as well as the current year of interest (four-tooth). Where data from previous years was considered, each year was modelled separately e.g., three models were created for the four-tooth analysis, the first using ewe lamb data as predictors, the second using two-tooth data, and the third considering data from the year of analysis, four-tooth. For all analyses, the individual ewe served as the unit of analysis, and the predictors were analysed on the dichotomous outcome “lambing fate” where each ewe was either “dead” or “alive” after the lambing season in the specific year of interest. Variables considered in the analyses were: weight and BCS at each visit, PD result (single or multiple-bearing), and, lactation status (wet or dry). Data analyses were conducted using RStudio version 2022.07.1.

To build the logistic models, a series of steps were followed. Firstly, univariable analyses were done and all variables that were associated with “lambing fate” (p ≤ 0.3) were considered for inclusion in the multivariable model. The predictor of “cohort” was included in each model, to account for differences between the four cohorts of ewe (Farm A 2010-born, Farm A 2011-born, Farm B and Farm C). Before inclusion in the multivariable model the categorical variables underwent an evaluation of their levels and distribution based on one-way tables. Next, two-way tables were constructed pairwise to assess the relationships between the variables. Subsequently, a backwards elimination procedure was implemented where the variable with the highest p-value was removed. A significant association was defined as p ≤ 0.05. If the removal of a variable resulted in an alteration of the estimates of the other variables in the model by > 15%, the removed variable was regarded as a confounder and was therefore retained in the model. Thereafter, variables that were not included in the multivariable analysis (p > 0.3) were added to the model one at a time. In accordance with the previous approach, any variable that resulted in a proportionate change of 15% or more in the estimates of any other predictor was considered a confounder and was thus retained in the model. If two or more variables in the model were highly correlated (such as the weight and BCS variables for different periods within the same year), one variable of each type was selected based on biological relevance and included in the final model. To evaluate potential interactions between variables, two-way interaction terms were added to the model one by one and their significance was assessed using the likelihood-ratio test. This forward procedure was repeated until only significant interactions remained in the final model. From the final models, odds ratios (OR), with 95% CI and their corresponding p-values, were reported.

## Results

### Ewe lamb (7 to 8-months-old at breeding) mortality

There were 11,143 ewe lambs retained for lambing, of which 819 (7.3%) died between set-stocking and weaning. Cohort, PD weight and set-stocking BCS were included in the final multivariable model. Heavier ewe lambs were less likely to die during lambing (Table 1), as were those with greater BCS (Table 1).

**Table 1:** Results of the final multivariable model, using ewe lamb predictors, showing the OR (95% CI) for risk of ewe lamb (7 to 8-months-old at breeding) mortality between set-stocking and weaning

Variable	Category	Odds ratio	95% CI	p-value
Cohort				
	Farm A 2010-born	1	-	-
	Farm A 2011-born	1.812	1.512–2.171	< 0.001
	Farm B	0.582	0.442–0.766	< 0.001
	Farm C	0.561	0.316–0.996	0.048
Ewe lamb PD weight		0.978	0.961–0.995	0.013
Ewe lamb set-stocking BCS				
	2.0	1	-	-
	2.5	0.589	0.405–0.855	0.005
	3.0	0.588	0.401–0.863	0.007
	3.5	0.565	0.348–0.918	0.021
	4.0	0.670	0.318–1.413	0.293

### Two-tooth (18-months-old at breeding) mortality

There were 8,912 two-tooth ewes retained for lambing, of which 561 (6.3%) died between PD and weaning. There was no association between weight, BCS or reproductive performance, as a ewe lamb, and two-tooth mortality risk; hence no data are shown. Cohort, two-tooth PD weight, two-tooth PD BCS and the interaction between weight and BCS were included in the final multivariable model. At the average weight, there was no statistically significant effect of BCS (Table 2). For two-tooth ewes that were BCS 2.0 at PD, increasing weight reduced risk of mortality during lambing; however, for ewes with a PD BCS > 2.0, there was no statistically significant effect from increasing weight (Table 2).

**Table 2:** Results of the final multivariable model, using two-tooth (18-months-old at breeding) predictors, showing the OR (95% CI) for risk of two-tooth mortality between pregnancy diagnosis (PD) and weaning

Variable	Category	Odds ratio	95% CI	p-value
Cohort				
	Farm A 2010-born	1	-	-
	Farm A 2011-born	0.833	0.664–1.045	0.114
	Farm B	0.422	0.318–0.559	< 0.001
	Farm C	0.049	0.015–0.153	< 0.001
Effect of BCS at average weight^1^				
Two-tooth PD BCS				
	2	1	-	-
	2.5	1.069	0.695–1.646	0.760
	3	0.874	0.540–1.415	0.585
	≥ 3.5	0.846	0.339–2.108	0.719
Effect of weight at different BCS^2^				
Two-tooth PD BCS				
	2.0	0.899	0.857–0.944	< 0.001
	2.5	1.006	0.982–1.030	0.641
	3.0	1.029	0.996–1.063	0.085
	≥ 3.5	1.044	0.960–1.134	0.313

^1^There was a significant interaction between weight and BCS. Therefore, this shows, for a ewe of average weight, the effect of BCS

^2^There was a significant interaction between weight and BCS. Therefore, this shows, for each BCS, the effect of increasing the weight by 1 kg

### Four-tooth (30-months-old at breeding) mortality

There were 6,627 four-tooth ewes retained for lambing, of which 458 (6.9%) died between PD and weaning. Three models were created, as ewe lamb, two-tooth and four-tooth variables were associated with risk of four-tooth mortality over lambing. Four-tooth mortality was associated with ewe lamb weaning weight (when ewes were approximately 15-months-old) and reproductive status (wet vs. dry at weaning). Four-tooth ewes that were heavier as ewe lambs at weaning were less likely to die during lambing although the effect was small (OR 0.974; 95% CI 0.955–0.994; p = 0.011), while those that failed to rear a lamb as a ewe lamb (i.e., dry at ewe lamb weaning) were more likely to die (OR 1.459; 95%CI 1.050–2.028; p = 0.024) compared to those that reared at least one lamb.

Mortality as a four-tooth was impacted by two-tooth weaning weight, mating BCS and the interaction between these, and reproductive status as a two-tooth (wet vs. dry at weaning) (Table 3). Ewes that were BCS 2.5 or 3.0 as a two-tooth were less likely to die than those with BCS 2.0 (Table 3). Additionally, for ewes that were BCS 2.5 or 3.0 as a two-tooth, increasing weight reduced risk of four-tooth mortality; however, for ewes with BCS > 3.0 there was no effect from increasing weight (Table 3). Ewes that failed to rear a lamb as a two-tooth (i.e., dry at two-tooth weaning) were more likely to die during four-tooth lambing compared to those that reared at least one lamb (wet) (Table 3).

**Table 3:** Results of the final multivariable model, using two-tooth (18-months-old at breeding) predictors, showing the OR (95% CI) for risk of four-tooth (30-months old at breeding) mortality between pregnancy diagnosis (PD) and weaning

Variable	Category	Odds ratio	95% CI	p-value
Cohort				
	Farm A 2010-born	1	-	-
	Farm A 2011-born	0.535	0.407–0.703	< 0.001
	Farm B	0.461	0.359–0.592	< 0.001
Wet vs. Dry at weaning				
	Wet	1	-	-
	Dry	1.949	1.082–3.513	0.026
Effect of BCS at average weight^1^				
Two-tooth mating BCS				
	2.0	1	-	-
	2.5	0.588	0.354–0.977	0.040
	3.0	0.501	0.302–0.831	0.007
	3.5	0.760	0.425–1.358	0.354
	4.0	0.508	0.212–1.218	0.129
Effect of weight at different BCS^2^				
Two-tooth mating BCS				
	2.0	1.069	0.989–1.156	0.091
	2.5	0.963	0.937–0.990	0.007
	3.0	0.958	0.933–0.983	0.001
	3.5	0.984	0.936–1.035	0.537
	4.0	1.024	0.944–1.111	0.566

^1^There was a significant interaction between weight and BCS. Therefore, this shows, for a ewe of average weight, the effect of BCS

^2^There was a significant interaction between weight and BCS. Therefore, this shows, for each BCS, the effect of increasing the weight by 1 kg

Mortality as a four-tooth was impacted by four-tooth PD BCS and PD weight and PD result (litter size) and the interaction between weight and PD result (Table 4). Ewes with BCS 2.5 or 3.0 at four-tooth PD were less likely to die compared to ewes with BCS 2.0 (Table 4). Multiple bearing ewes were more likely to die compared to single-bearing ewes, while single bearing ewes with increasing weight were less likely to die, but this relationship was not detected for multiple-bearing ewes (Table 4).

**Table 4:** Results of the final multivariable model, using four-tooth (30-months-old at breeding) predictors, showing the OR (95% CI) for risk of four-tooth mortality between pregnancy diagnosis (PD) and weaning

Variable	Category	Odds ratio	95% CI	p-value
Cohort				
	Farm A 2010-born	1	-	-
	Farm A 2011-born	0.537	0.404–0.712	< 0.001
	Farm B	0.528	0.417–0.667	< 0.001
Four-tooth PD BCS				
	2.0	1	-	-
	2.5	0.512	0.361–0.725	< 0.001
	3.0	0.534	0.369–0.772	< 0.001
	3.5	0.801	0.489–1.312	0.378
Effect of PD result at average weight^1^				
Litter size (PD result)				
	Single-bearing	1	-	-
	Multiple-bearing	1.487	1.160–1.906	0.002
Effect of weight at different PD results^2^				
Litter size (PD result)				
	Single-bearing	0.918	0.886–0.952	< 0.001
	Multiple-bearing	0.960	0.336–2.745	0.939

^1^There was a significant interaction between weight and PD result. Therefore, this shows, for a ewe of average weight, the effect of PD result (litter size)

^2^There was a significant interaction between PD (litter size) and weight. Therefore, this shows, for each litter size (single- vs. multiple-bearing), the effect of increasing the weight by 1 kg

### Six-tooth (42-months-old at breeding) mortality

There were 5,126 six-tooth ewes retained for lambing, of which 342 (6.7%) died between PD and weaning. Three models were created, as ewe lamb, four-tooth and six-tooth variables were associated with risk of six-tooth mortality over lambing; however, there was no relationship between two-tooth variables and six-tooth mortality risk. BCS as a ewe lamb and a four-tooth was associated with mortality over lambing as a six-tooth. Increased ewe lamb set-stocking BCS was associated with reduced risk of mortality during six-tooth lambing (Supplementary Table 1). Similarly, increasing four-tooth weaning BCS was associated with reduced risk of mortality during the six-tooth lambing period (Supplementary Table 2).

Mortality as a six-tooth was impacted by six-tooth PD result (litter size), PD BCS, PD weight and the interaction between BCS and weight (Table 5). Multiple-bearing ewes were more likely to die than single-bearing ewes (Table 5). Increasing PD BCS was associated with reduced risk of mortality over lambing (Table 5). Additionally, for ewes that were BCS 2.0 at PD, increasing weight reduced risk of mortality during lambing; however, for ewes with a PD BCS > 2.0, there was no statistically significant effect observed with increasing weight (Table 5).

**Table 5:** Results of the final multivariable model, using six-tooth (42-months-old at breeding) predictors, showing the OR (95% CI) for risk of six-tooth mortality between pregnancy diagnosis (PD) and weaning

Variable	Category	Odds ratio	95% CI	p-value
Cohort				
	Farm A 2010-born	1	-	-
	Farm A 2011-born	0.799	0.586–1.091	0.158
	Farm B	0.555	0.411–0.750	< 0.001
Litter size (PD result)				
	Single-bearing	1	-	-
	Multiple-bearing	1.641	1.224–2.199	< 0.001
Effect of BCS at average weight^1^				
Six-tooth PD BCS				
	2.0	1	-	-
	2.5	0.259	0.125–0.536	< 0.001
	3.0	0.208	0.104–0.417	< 0.001
	3.5	0.218	0.095–0.500	< 0.001
Effect of weight at different BCS^2^				
Six-tooth PD BCS				
	2.0	0.847	0.787–0.911	< 0.001
	2.5	0.962	0.923–1.004	0.075
	3.0	1.013	0.979–1.047	0.461
	3.5	0.997	0.932–1.067	0.936

^1^There was a significant interaction between weight and BCS. Therefore, this shows, for a ewe of average weight, the effect of BCS

^2^There was a significant interaction between weight and BCS. Therefore, this shows, for each BCS, the effect of increasing the weight by 1 kg

### Mixed-age (54-months-old at breeding) mortality

There were 3,840 mixed-age ewes retained for lambing, of which 240 (6.3%) died between PD and weaning. Four models were created, as ewe lamb, two-tooth, four-tooth and mixed-age variables were associated with risk of mixed-age mortality over lambing; however, there was no relationship between six-tooth variables and mixed-age mortality risk. Ewes that were dry as a ewe lamb (didn’t rear a lamb to weaning) had lower risk of mortality as a mixed-age ewe (OR 0.625; 95%CI 0.396–0.985; p = 0.043). Ewes that were multiple-bearing as two-tooths had greater risk of mortality as a mixed-age ewe (OR 1.675; 95%CI 1.243–2.256; p < 0.001) compared to those that were single-bearing as two-tooths. BCS at mating as a two-tooth and four-tooth was associated with mortality, with increasing BCS associated with reduced risk of mortality during mixed-age lambing (Supplementary Tables 3 and 4). Mixed-age ewe mortality was associated with mixed-age ewe PD BCS, with risk of mortality decreasing for ewes with increasing BCS (Table 6).

**Table 6:** Results of the final multivariable model, using mixed-age (54-months-old at breeding) predictors, showing the OR (95% CI) for risk of mixed-age mortality between pregnancy diagnosis (PD) and weaning

Variable	Category	Odds ratio	95% CI	p-value
Mixed-age PD BCS				
	2.0	1	-	-
	2.5	0.159	0.092–0.274	< 0.001
	3.0	0.098	0.058–0.163	< 0.001
	3.5	0.120	0.059–0.242	< 0.001

## Discussion

In this study, in the approximately 26-week period from PD to weaning, ewe mortality incidence ranged from 6.3 to 6.9% for two-tooth (18-months-old at breeding) to mixed-age (54-months-old at breeding) ewes. For ewe lambs (7 to 8-months-old at breeding), mortality incidence was 7.3% in the approximately 18-week period from set-stocking to weaning. These rates are comparable to those previously reported under pasture-based conditions in New Zealand by [8, 18, 19], with reported rates of 2.5 to 7.5%, 9.6% and 6.8%, respectively, for similar time-periods. [20] reported a 2.5% mortality rate in ewe lambs over a 6-week period coinciding with lambing, while a further 1.8% had an assisted birth; it is unknown if those ewe lambs would have died or survived if unassisted. In a comparable time-period in Australian flocks (PD to tailing or weaning), mortality rates of 0 to 13.7% have been reported [4, 9, 10, 13]; however, both [13] and [9] used farmer-reported mortality data, which may be inaccurate and potentially under-estimate ewe mortality [14].

Ewe mortality is costly, with economic costs such as loss of productivity and replacement costs [2, 3, 7, 21, 22], welfare costs associated with on-farm ewe death [4, 5] and reputational costs associated with public and consumer perception of ewe death [23, 24]. If ewes die during the pregnancy and lambing period this also represents opportunity cost, as these ewes have been fed since weaning the previous year (or selected as replacements in the case of ewe lambs), yet there is no economic return, besides wool value, and there is also the concurrent loss of their lambs. Additionally, in extensive New Zealand flocks, ewe mortality has been attributed as the cause of 11 to 21% of lamb mortalities [18, 20], reducing the number of lambs available for sale. If ewes at increased risk are identified before this time-period, then flock owners have the option of selectively culling these ewes, or alternatively, increase levels of potential interventions to reduce the likelihood of mortality, thereby improving individual ewe and whole flock productivity, profitability and welfare outcomes.

The greater risk of mortality during pregnancy and lambing in poor BCS ewes was a key finding in this study, with poor BCS a consistent risk factor. Recently, [25] reported 8–12% of ewes in New Zealand flocks are estimated to be BCS 2.0 or less at PD or set-stocking. Therefore, at a national level, there are reasonably large numbers of ewes that are at increased risk of mortality due to their poor BCS. Research has also demonstrated the importance of ewe BCS as a risk factor for annual ewe mortality in New Zealand [7, 8], Australia [26] and Ireland [27]; with poor BCS ewes, particularly those with BCS ≤ 2.0, having the greatest risk of mortality. If farmers have poorer BCS ewes in their flock, there will likely be benefit from identifying these ewes and preferentially feeding them.

It is also important to note, as has been reported with BCS and other production traits [28], the relationship between BCS, weight and mortality was not always linear. Poor BCS ewes with increasing weight had a reduced risk of mortality during lambing, but this was not observed with better BCS ewes. There was also variation in the magnitude of the OR with different ages of ewe. In general, the older the ewes were, the greater the impact that poor BCS had on mortality. This suggests that there may be further benefit gained by targeted interventions towards older, poorer BCS ewes.

Wastage of younger ewes within a flock is particularly costly to farmers as there are no productive or economic benefits if ewe lambs die before they rear lambs for sale [7]. In this study, heavier ewe lambs were less likely to die, emphasizing the importance of following recommendations around minimum breeding weight and weight gain during pregnancy. Ewe lambs need to be well grown prior to breeding [29] and then fed well to gain weight throughout pregnancy [30] in lactation and post-weaning [29] to both improve their productivity [29, 31] and reduce their risk of premature culling and mortality [7].

In the present study the cause of ewe death was not established, however results of previous studies allow us to hypothesise about the most likely causes of mortality. The most commonly reported causes of ewe death in extensively managed pastoral flocks during the pregnancy and lambing period include dystocia, vaginal prolapse, metabolic disorders and casting (ewe recumbent on back and unable to rise) [8, 10, 12, 19]. In this study, multiple bearing ewes were generally at greater risk of mortality than single bearing ewes. Diseases such as vaginal prolapse, metabolic disorders and casting are more likely to affect multiple-bearing ewes [8, 32–34], and litter size has been reported as a potential risk factor for ewe mortality [9]. Combined, this suggests that there may be benefit from farmers monitoring their multiple-bearing ewes more intensively during the pregnancy and lambing period, enabling rapid intervention when issues are identified.

Ewe mortality during the pregnancy and lambing period are issues for sheep farmers globally [2]. In New Zealand, flocks are typically managed under an ‘easy-care’ lambing system with minimal shepherding or human intervention, with ewes purposely selected over time to suit this extensive management [35, 36]. Hence, there may be differences in mortality rates and causes between this type of system and intensive pasture-based or indoor lambing systems such as those in the United Kingdom and Europe. However, even in these more intensively shepherded flocks ewe mortality rates increase during lambing [37], and dystocia, vaginal prolapse and metabolic disorders remain key causes of death during this period [33, 37].

In this study, most of the ewes that were classified as ‘dead’ were missing, rather than being identified as dead, and necropsies were not done on dead ewes to determine cause of death. It is possible that some of these missing ewes may have been incorrectly classified as dead; and instead had lost tags, were in incorrect mobs, or were culled from the flock without being recorded. However, the frequency of visits that coincided with key whole-flock management times (e.g., all ewes on the farm, not just study ewes, were observed by the flock manager/shepherds prior to breeding, during PD and at weaning), combined with the longitudinal nature of the study, mean misclassification is unlikely. The degree to which the EID tags may have been lost in the present study is unknown. However, EID ear-tag losses in Europe (where electronic identification of small ruminants is mandatory) are reported to be less than 4% per annum [38]. Difficulties investigating ewe mortality rates and causes have been previously described, with other authors reporting challenges identifying all dead ewes even with intensive daily observations [8]. Additionally, in instances where cause of death has been investigated, a large proportion (as many as 20–30%) are still classified as unknown [39].

## Conclusion

This study describes mortality during the pregnancy and lambing period in different aged ewes, with incidence ranging from 6.3 to 7.3% during this period. Heavier ewe lambs were less likely to die during the lambing period, as were those with greater BCS, emphasizing the importance of following recommendations around ewe lamb management prior to breeding and throughout pregnancy and lactation, to not only improve productivity, but also reduce the risk of death. In subsequent years, BCS was a predictor of ewe death, with odds of mortality greatest for ewes < BCS 2.5. Additionally, for poorer BCS ewes, increasing weight reduced risk of mortality, but there was no effect of increasing weight in greater BCS ewes. Combined, this information can be used by flock managers to identify ewes that are at greatest risk, to either intervene to reduce risk, or selectively cull them from the flock. For instance, there is likely benefit for farmers identifying poorer BCS ewes within the flock and preferentially feeding them. There may also be benefit from monitoring multiple-bearing ewes more intensively during pregnancy and lambing, as these ewes were generally at greater risk of mortality than single bearing ewes. Overall, the results of this study could be used to reduce ewe mortality during pregnancy and lambing, improving flock productivity and profitability and individual ewe welfare.

### Electronic supplementary material

Below is the link to the electronic supplementary material.

Supplementary Material 1

## Data Availability

The datasets are not publicly available as they contain information about the participating farms and flocks. However, data is available from the corresponding author on reasonable request.
