# Supplementary material for: Identification of risk factors for ewe mortality during the pregnancy and lambing period in extensively managed flocks
Source: BMC Vet Res. 2023 Dec 6;19:257. doi: 10.1186/s12917-023-03822-x (PMC10698936; doi:10.1186/s12917-023-03822-x)
Supplement: Supplementary file 1 — Supplementary Material 1 [file 12917_2023_3822_MOESM1_ESM.docx]

**Supplementary Table 1**

Results of the final multivariable model, using ewe lamb predictors, showing the OR (95% CI) for risk of six-tooth mortality during the lambing period

| Variable | Category | Odds ratio | 95% CI | p-value |
| --- | --- | --- | --- | --- |
| Ewe lamb set-stocking BCS |  |  |  |  |
|  | 2.0 | 1 | - | - |
|  | 2.5 | 0.437 | 0.237 - 0.806 | 0.008 |
|  | 3.0 | 0.378 | 0.205 - 0.699 | 0.002 |
|  | 3.5 | 0.448 | 0.223 - 0.901 | 0.024 |

**Supplementary Table 2**

Results of the final multivariable model, using four-tooth predictors, showing the OR (95% CI) for risk of six-tooth mortality during the lambing period

| Variable | Category | Odds ratio | 95% CI | p-value |
| --- | --- | --- | --- | --- |
| Four-tooth weaning BCS |  |  |  |  |
|  | 2.0 | 1 | - | - |
|  | 2.5 | 0.810 | 0.466 - 1.408 | 0.455 |
|  | 3.0 | 0.571 | 0.329 - 0.993 | 0.047 |
|  | 3.5 | 0.490 | 0.267 - 0.898 | 0.021 |
|  | 4.0 | 0.554 | 0.243 - 1.262 | 0.160 |

**Supplementary Table 3**

Results of the final multivariable model, using two-tooth predictors, showing the OR (95% CI) for risk of mixed-age mortality during the lambing period

| Variable | Category | Odds ratio | 95% CI | p-value |
| --- | --- | --- | --- | --- |
| Two-tooth mating BCS |  |  |  |  |
|  | 2.0 | 1 | - | - |
|  | 2.5 | 0.515 | 0.272 - 0.977 | 0.042 |
|  | 3.0 | 0.482 | 0.251 - 0.924 | 0.028 |
|  | 3.5 | 0.518 | 0.225 - 1.193 | 0.123 |
|  | 4.0 | 0.160 | 0.033 - 0.782 | 0.024 |
|  |  |  |  |  |
| Litter size (PD result) |  |  |  |  |
|  | Single-bearing | 1 | - | - |
|  | Multiple-bearing | 1.675 | 1.243 - 2.256 | < 0.001 |

**Supplementary Table 4**

Results of the final multivariable model, using four-tooth predictors, showing the OR (95% CI) for risk of mixed-age mortality during the lambing period

| Variable | Category | Odds ratio | 95% CI | p-value |
| --- | --- | --- | --- | --- |
| Four-tooth mating BCS |  |  |  |  |
|  | 2.0 | 1 | - | - |
|  | 2.5 | 0.484 | 0.259 - 0.904 | 0.023 |
|  | 3.0 | 0.420 | 0.225 - 0.787 | 0.007 |
|  | 3.5 | 0.370 | 0.163 - 0.840 | 0.017 |
